# Co-administration of Intravenous Drugs: Rapidly Troubleshooting the Solid Form Composition of a Precipitate in a Multi-drug Mixture Using On-Site Raman Spectroscopy

**DOI:** 10.1021/acs.molpharmaceut.2c00983

**Published:** 2023-05-11

**Authors:** Niklas Nilsson, Katerina Nezvalova-Henriksen, Johan P. Bøtker, Niels Højmark Andersen, Bjarke Strøm Larsen, Jukka Rantanen, Ingunn Tho, Jørgen Brustugun

**Affiliations:** † Department of Pharmacy, University of Oslo, Oslo 0316, Norway; ‡ Oslo University Hospital and Oslo Hospital Pharmacy, Hospital Pharmacies Enterprise, South-Eastern Norway, Oslo 0372, Norway; § Department of Pharmacy, University of Copenhagen, Copenhagen 2100, Denmark; ∥ Department of Chemistry, University of Oslo, Oslo 0315, Norway

**Keywords:** co-infusion, physical incompatibility, safe administration, emboli, patient safety, precipitate identification

## Abstract

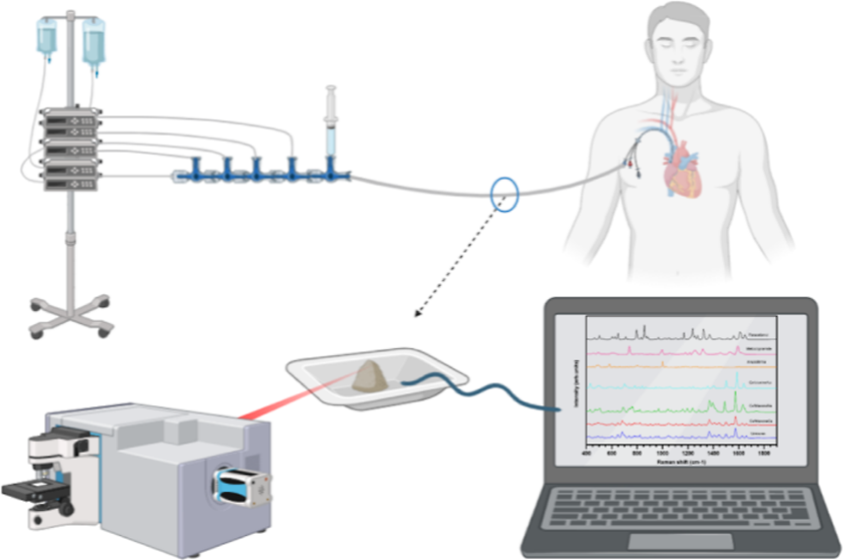

Intravenous drugs are often co-administrated in the same intravenous catheter line due to which compatibility issues, such as complex precipitation processes in the catheter line, may occur. A well-known example that led to several neonatal deaths is the precipitation due to co-administration of ceftriaxone- and calcium-containing solutions. The current study is exploring the applicability of Raman spectroscopy for testing intravenous drug compatibility in hospital settings. The precipitation of ceftriaxone calcium was used as a model system and explored in several multi-drug mixtures containing both structurally similar and clinically relevant drugs for co-infusion. Equal molar concentrations of solutions containing ceftriaxone and calcium chloride dihydrate were mixed with solutions of cefotaxime, ampicillin, paracetamol, and metoclopramide. The precipitate formed was collected as an “unknown” material, dried, and analyzed. Several solid-state analytical methods, including X-ray powder diffraction, Raman spectroscopy, and thermogravimetric analysis, were used to characterize the precipitate. Raman microscopy was used to investigate the identity of single sub-visual particles precipitated from a mixture of ceftriaxone, cefotaxime, and calcium chloride. X-ray powder diffraction suggested that the precipitate was partially crystalline; however, the identity of the solid form of the precipitate could not be confirmed with this standard method. Raman spectroscopy combined with multi-variate analyses (principal component analysis and soft independent modelling class analogy) enabled the correct detection and identification of the precipitate as ceftriaxone calcium. Raman microscopy enabled the identification of ceftriaxone calcium single particles of sub-visual size (around 25 μm), which is in the size range that may occlude capillaries. This study indicates that Raman spectroscopy is a promising approach for supporting clinical decisions and especially for compatibility assessments of drug infusions in hospital settings.

## 1 Introduction

Therapy regimens in hospital wards are growing in complexity.^1^ According to Hecq et al., 32 and 13% of patients are prescribed three or four injectable drugs to be administered at the same time, respectively.^2^ These drugs must be delivered to the patient within a limited time frame, frequently as continuous infusions, and through a limited number of intravenous (i.v.) lines. Even though multiple lumen central venous catheters (CVCs) are available, each lumen adds to the diameter of the catheter. The limitations in venous access are most severe in the neonatal intensive care unit (NICU) and pediatric intensive care unit (PICU), where patients can only tolerate the insertion of a single-, double-, or maximally a triple-lumen CVC.^3,4^ When an increasing number of drugs are required to be administered through a limited number of i.v. catheter ports (lumen), the reality in the clinic is that the drugs are co-administered using multi-connectors into the same port. The problem here is that the infused solutions that meet each other in the port may not be compatible; chemical degradation or precipitation may occur. The existing compatibility studies and databases, such as Trissel’s Handbook on Injectable Drugs, IV compatibility via Micromedex and Stabilis, cover for the most part pairs of drugs.^5−7^ For combinations of three or more infusions, the amount of literature is very limited, leaving the involved healthcare personnel (clinician, nurse, and clinical pharmacist) without any evidence-based knowledge. With the increasing number of drugs being combined, the detection of incompatibility (e.g., precipitation) is still as important as before; however, identification of the precipitated species (often referred to as a “white powder”, “clouding”, or “slush” in the line) is increasingly important. It is vital to identify the composition of a precipitate in a multi-drug combination to provide information regarding which drug has not reached the patient or possibly reached the patient in its inactive and/or precipitated form and to suggest how the combination of drugs should be ideally administered. Infusing particles of uncontrolled size poses a safety risk to the patient. Infused particles can clog the line, accumulate in organs, or block tiny capillaries causing embolism.^8^ A strategy to solve this delivery challenge is to rapidly identify the drug to be eliminated from co-administration via multi-connectors and be prioritized for a separate lumen.

Traditionally, the chemical stability of drugs for infusion, in our case after co-administration via the same port, has been studied using quantitative methods like high-performance liquid chromatography,^9−13^ while physical compatibility (e.g., precipitation, color change, and oil droplet growth in an emulsion) frequently was relying on visual observations, aided by microscopy or Tyndall beam.^14−16^ These approaches have methodological and practical shortcomings not least when combinations of three or more drugs are of interest. Developing validated methods for multiple component solutions demands time and resources, and relying on visual control for detection of precipitates does not cover sub-visible particles neither does it provide the solid form identity of the precipitate. The current methods for assessment of physical y-site compatibility include pH and turbidity measurements, sub-visual particle counting, osmolality, and if one of the components are or contain a parenteral lipid emulsion also mean droplet diameter measurement, and estimation of percentage of large diameter droplets (>5 μm, PFAT5).^17−21^ However, in neither of these methods, the identity of the precipitate can be determined beyond theoretical predictions based on solubility and pH. Raman spectroscopy could potentially redeem some of the challenges met with classical compatibility testing,^16,17,22^ extending the limits of what can be achieved. Raman spectroscopy is a method based on scattering of monochromatic light (laser), where a tiny part of light interacts with the sample, resulting in a Raman spectrum according to vibrational transitions in the illuminated sample material. The method provides, in broader sense, similar information as infrared spectroscopy, but with the advantage of being non-invasive and suitable for samples containing water, even analyzing a sample in an aqueous suspension.^23^ Additionally, Raman spectroscopy is sensitive to interactions between solutes and solvents, i.e., local molecular surroundings as well as conformational changes and aggregation.^24,25^ This implies that the Raman spectra of the same compound may differ depending on the chosen conditions.^24,26^ As a summary, Raman spectra can be obtained from solids, slurries, and solutions, and it is a rapid and non-invasive technique without the need of sample preparation or very little sample preparation. Raman is well established for process analytics, and there is an obvious similarity with the challenges related to identification of the solid form in the hospital setting when compared with analysis of moving matter in process environment.^27^ As drug products for infusions and injections are often simple solutions of drugs in water, with few and simple excipients, Raman spectroscopy could prove a powerful tool for studying compatibility in complex infusion regimens, especially precipitations originating from incompatibilities. Recent development of handheld instrumentation^28^ opens up an attractive opportunity to bring vibrational spectroscopy into a hospital setting.

Incompatibility in hospital settings, as mentioned above, occurs often, and consequences can be serious and unacceptable. A documented example of a fatal outcome is the concomitant administration of the antibiotic ceftriaxone with calcium-containing products in neonates.^29^ This co-administration led to the formation of solid precipitates that were detected in vascular beds during autopsy, mostly in lungs.^29−31^ In this study, the ceftriaxone calcium precipitation serves as a *model system* to explore the potential of Raman spectroscopy for the identification of precipitate in a multi-drug mixture relevant in a future hospital setting.

## 2 Materials and Methods

### 2.1 Materials

The drug products for testing ceftriaxone, cefotaxime, ampicillin, paracetamol, and metoclopramide were purchased from the local hospital pharmacy. The marketed drug products were used as raw materials, instead of the available analytical grade drugs, in order to explore the relevance of Raman spectroscopy in compatibility studies in a hospital setting. Since the prescribed drug products also contain excipients, their impact needs to be part of the study design.^32^ Calcium chloride dihydrate powder was purchased from Sigma-Aldrich (St. Louis, MO, USA). Table 1 provides an overview of the drug products used in the study.

**Table 1:** Overview of the Marketed Drug Formulations Used, Their Excipients, Molecular Weights (MW), and Final Concentrations

drug formulation (manufacturer) dosage form	excipients^a^	MW (g/mol)	final concentration
ceftriaxone sodium (Stragen) powder, 2 g		554.58	90 mM
cefotaxime sodium (Stragen) powder, 1 g		477.50	90 mM
ampicillin sodium (STADA) powder, 1 g		349.40	90 mM
paracetamol (Fresenius-Kabi), solution for injection	cysteine, mannitol, and water for injection	151.16	10 mg/mL (≈70 mM)
metoclopramide hydrochloride (Oripharm Healthcare), solution for injection	sodium chloride, sodium pyrosulfate, and water for injection	336.26	5 mg/mL (≈20 mM)
calcium chloride dihydrate (Sigma) powder		147.01	90 mM
glucose monohydrate (B. Braun), solution for infusion		180.16	50 mg/mL (≈300 mM)

a From the summary of product characteristics.

### 2.2 Sample Preparation

Solid samples of all powdered drug products were used as the raw material (Table 1). As paracetamol and metoclopramide hydrochloride were received as solutions, the solid samples of these drug products were obtained by drying the solution at ambient temperature overnight.

Stock solutions of ceftriaxone sodium (ceftriaxoneNa), cefotaxime sodium (cefotaximeNa), ampicillin sodium (ampicillinNa), and calcium chloride dihydrate were prepared individually by diluting the dry substance with purified water from a Milli-Q deionization unit (Millipore, Bedford, MA, USA; denoted as MQ-water) to a concentration of 90 mM. Paracetamol and metoclopramide hydrochloride were used undiluted as received from the manufacturer.

Since ceftriaxone calcium (ceftriaxoneCa) was not commercially available, it was produced by precipitation after mixing equal volumes of equimolar concentrations of ceftriaxone sodium and calcium chloride dihydrate. After 90 min, the supernatant was removed, the precipitate was washed with 2 mL of MQ-water and centrifuged for 2 min at 13,900 rpm (room temperature), and the supernatant was then removed by careful pipetting. The sample was dried for 4 h in an incubator (Termaks, Bergen, Norway) at 40 °C, followed by overnight drying at ambient temperature.

In order to mimic the clinical scenario of multiple drug co-administration in the same i.v. catheter line, the “unknown” precipitate was prepared by mixing equimolars of stock solutions of ceftriaxoneNa, cefotaximeNa, ampicillinNa, and calcium chloride dihydrate and by adding metoclopramide and paracetamol in equal volumes to gain a mixture of more different drugs (Table 1). After 2.5 h, 2 mL of the suspension with the “unknown” precipitate was transferred to an Eppendorf tube. The supernatant was removed. The sample was washed once with 2 mL of MQ-water and centrifuged for 2 min at 13,900 rpm (room temperature), and the supernatant was removed by careful pipetting. The sample was dried to apparent dryness in an incubator at 40 °C for 12 h.

A blank reference (i.e., without ceftriaxoneNa) was prepared from the stock solutions of cefotaximeNa, ampicillinNa, metoclopramide, and paracetamol with calcium chloride dihydrate.

### 2.3 Characterization of Solid Raw Materials and Mixtures

#### 2.3.1 Visual Inspection and Polarized Light Microscopy

For a first quick assessment of whether mixing resulted in particle precipitation, the all drug-mixed sample and the blank reference were inspected visually. Within 2 h after mixing of drug solutions, the samples were examined using a focused light beam (630–650 nm, P 3010 RoHS, Chongqing, China) against a black background to check for any Tyndall effect (i.e., visible coherent laser line through the sample). A droplet of the sample was also examined using polarized light microscopy (Leica, Wetzlar, Germany).

#### 2.3.2 X-ray Powder Diffraction

The powder samples of the solid raw materials including ceftriaxoneCa were examined. For these analyses, paracetamol and metoclopramide were not included since they were received as solutions. Instead, a powdered sample of paracetamol (Fagron, Barcelona, Spain) was included for reference. X-ray powder diffraction (XRPD) analysis of all samples was performed using an X’Pert PRO X-ray diffractometer (PANalytical, Almelo, The Netherlands) using Cu Kα radiation (λ = 1.541 Å), with an angular increment of 0.04/s and a count time of 2 s. The acceleration voltage and current were 45 kV and 40 mA, respectively. Diffractograms were normalized and stacked on an arbitrary scale in order to allow qualitative comparison.

#### 2.3.3 Thermogravimetric Analysis

Powdered samples of ceftriaxoneNa, ceftriaxoneCa, calcium chloride dihydrate, and “unknown” sample were examined using a Discovery Thermogravimetric Analyzer from TA-Instruments-Waters LCC (New Castle, DE, USA). The samples were loaded in a flame-cleaned platinum pan and heated at 10 °C/min from ambient temperature to 250 °C. The weight loss was analyzed using the Trios software version 5.5.1 from TA-Instruments-Waters LCC (New Castle, DE, USA). Weight loss as a function of temperature represents the loss of water/solvent (sorbed or crystal water/hydrates/solvates) or degradation of the compound.

#### 2.3.4 Scanning Electron Microscopy

Solid powder samples of all raw materials, ceftriaxoneCa, and the “unknown” precipitated sample were examined. The cross-sectional surface area of the sample as it was removed from the Eppendorf vials was imaged. The samples were mounted on carbon adhesive tape, sputter-coated with gold under argon vacuum with a Sputter Coater 108auto (Cressington Scientific Instruments Ltd., Watford, UK), and investigated with a Hitachi Analytical TableTop SEM TM3030 scanning electron microscope (Hitachi High-Technologies Europe GmbH, Krefeld, Germany). Micrographs were taken at different magnifications: 200-fold magnification to obtain overview micrographs and 600–2000-fold magnification for detailed inspection.

### 2.4 Raman Spectroscopy

Two different Raman instruments at two different labs were used in this study; one is superior for averaging over larger powder samples, whereas the other is specialized for studies of single particles. Powder samples obtained from substantial precipitation events from a multi-drug mix were investigated and combined with multi-variate analysis to determine the identity of the “unknown” precipitate by various approaches (Sections 2.4.1–2.4.3). As a refinement, single particles were precipitated and captured on a filter, and the identity of single, sub-visual sized particles of “unknown” particles was proven (Section 2.4.4). Library spectra were captured from powdered raw materials of each drug product separately.

#### 2.4.1 Raman Spectra Acquisition and Processing of Powder Samples

All powder samples of raw materials as well as the “unknown” sample were placed on a glass (microscope) slide, and spectra were recorded using the Raman spectrometer Kaiser RXN1 Microprobe (Kaiser Optical Systems, Ann Arbor, MI, USA) with a PhaT-probe (Kaiser Optical Systems), controlled by HoloGRAMS software. The measurements were carried out using a laser source with a wavelength of 785 nm. The Raman shifts from 150 to 1900 cm^–1^ were acquired, each spectrum comprising 5801 data points. The laser spot size was 6 mm on the powder samples, and six exposures of 10 s each were averaged for each sample, giving a total exposure time of 60 s. Ten to 14 measurements were obtained for each sample. Baseline correction (weighted least-squares algorithm with a second-order polynomial) was performed to minimize the artefacts due to the fluorescence and differences in offset. The spectra were normalized and stacked on an arbitrary scale in order to allow qualitative comparison.

As a potential straightforward tool at point of care to be used to compare Raman spectra, Pearson correlation coefficients, *r*^2^, were directly calculated between the spectra of the individual raw materials (reference) and the “unknown” sample. It is recognized that this simple analysis of the spectra might miss key differences, e.g., peak position shifts. However, it can be utilized as a rapid troubleshooting tool to potentially identify the likely precipitating material and/or the parent compound. These correlation coefficients were calculated on the average spectrum made on the “unknown” sample against the average spectra of the individual raw materials, including ceftriaxoneCa, using the measured intensity for the same wavenumber in all the Raman spectra. This in essence assumes a linear relationship between the intensities measured for the same wavenumbers.

The calculated Pearson correlation coefficient is not sensitive to the relative intensity of the obtained spectra nor any zero-order baseline corrections but shows the correlation between intensity changes at the same wavenumbers, i.e., peak positions. These correlation coefficients were used to predict the identity of the “unknown” sample as well as the probability of origin. Correlation coefficients are reported as the proportion of explained variance between the “unknown” sample and raw material spectra, *r*^2^, and shown in percentage.

#### 2.4.2 Principal Component Analysis

All Raman spectra of the “unknown” and the individual raw materials (400–1900 cm^–1^) were pre-possessed using standard normal variate correction and mean cantering. For the comparison of the “unknown” precipitated samples and the library (reference) samples, a principal component analysis (PCA) was used (The Unscrambler X version 11, Camo ASA, Trondheim, Norway). The PCA transforms correlated variables into uncorrelated variables, which are called principal components (PCs). This helps to decrease the dimensionality in the data while keeping the variability in Raman spectral data. PC1 explains maximum variability in the data, while each successive PC explains the remaining variability. Grouping of samples was evaluated by carefully examining the scores and loading plots. Hotelling’s *T*^2^ statistics with the ellipse set at 5% significance was employed on the scores to identify the distance from the center of the model.^33^

#### 2.4.3 Classification by Soft Independent Modelling Class Analogy

To assign the “unknown” samples to one of the reference classes based on their Raman spectra, soft independent modelling class analogy (SIMCA) was applied.^33^ First, individual PCA models were made from the spectra of the individual raw material including ceftriaxoneCa (i.e., library spectra). Then, the spectra of the “unknown” sample were classified based on all the established PCA models. Based on the Coomans’ plot and the membership plot, the identity of the “unknown” sample could be determined at 95% significance level.^33^

#### 2.4.4 Identity of the Sub-visible Particles by Raman Microscopy

A second Raman spectrometer, a HORIBA Jobin-Yvon T64000 Raman Instrument equipped with a confocal microscope (Lille, France), was used to explore the detection and identification of sub-visual particles of ceftriaxoneCa in a more clinically relevant setup. CeftriaxoneNa was mixed with calcium chloride dihydrate in the presence of the structurally similar drug cefotaximeNa. The addition of calcium chloride was explored in mixing ratios and different addition rates in order to produce a precipitate that was not detectable by the naked eye but could be captured on a glass microfiber filter with a pore size of 1 μm (Whatman, GE Healthcare, UK). Prior to mixing, all drugs were diluted separately with isotonic glucose (50 mg/mL), each to a concentration of 90 mM. Ten parts (volume) of ceftriaxoneNa was mixed with 25 parts of cefotaximeNa and one part of calcium chloride dihydrate. Any precipitated particles were dried using suction filtration. Care was taken to capture the precipitate scarcely distributed on the filter. Several filters were prepared. The filter with the precipitated particles was placed under the Raman microscope, particles within the size range 5–50 μm was identified, and its Raman spectrum was recorded.

The spectra of individual particles on the filters were identified with the confocal microscope of the HORIBA Jobin-Yvon T64000 Raman Instrument. The spectrograph was equipped with a 300 groove/mm grating blazed at 600 nm combined with an entrance slit width of 100 μm, a 100 μm confocal pinhole, and a 785 nm Razoredge long-pass filter from Semrock. An open electrode 256 × 1024 CCD detector cooled to −130 °C was used. The microscope objective used was a 20× NIR type from Olympus. A Matisse tunable laser pumped with a Millennia SJ12 YVO4 532.1 nm laser running at 8 W resulted in a beam with 787 nm wavelength and a power of 500 mW. This was damped through several ND filters to a power of 6 mW measured at the sample. The spectra of the particles were averages of 10 spectra of 60 s acquisition. For the pure compounds, the spectra were produced by averaging 30 spectra with 10 s acquisition. All spectra were scale-calibrated against the spectra of paracetamol that was used as a reference compound.^34^ The background was subtracted by use of six- to eight-degree polynomia, and difficult spectral features stemming from the glass filter were subtracted by use of broad Gaussian functions. Normalized spectra were stacked on an arbitrary scale to allow qualitative comparison. In addition, the Pearson correlation coefficient was calculated between the spectrum of the “unknown” sample and the spectra of the raw materials in the same way as described in Section 2.4.1.

## 3 Results and Discussion

### 3.1 Visual Assessment of Multi-drug Mixtures

To explore the specificity of the setup, a multi-drug mixture was chosen that contained both structurally similar drugs (ceftriaxone sodium and calcium salt in addition to cefotaxime) and clinically relevant drugs that could be co-administered with the cephalosporins (ampicillin, paracetamol, and metoclopramide).

Mixing all the drugs, namely, ceftriaxoneNa, cefotaximeNa, ampicillinNa, paracetamol, metoclopramide, and calcium chloride dihydrate, in concentrations reported in Table 1, lead to massive precipitation. However, mixing all the same drugs and calcium chloride dihydrate but omitting ceftriaxoneNa in the solution yielded no precipitation. This was confirmed both visually using Tyndall beam and by microscopic observation. The finding suggests that the precipitate formed in the full six component mixture (designated as “unknown”) contains ceftriaxoneCa and, therefore, that the mixture without ceftriaxoneNa is a suitable blank reference.

### 3.2 Characterization of Solid Raw Materials and Mixtures (XRPD, TGA, and SEM)

The sample containing the precipitate from the six-component mixture was prepared with the intention of exploring Raman spectroscopic analysis for the identification of the “unknown”. Factors that could possibly influence this identification, like water content, solid form, or morphology, were characterized. Although Raman spectroscopy is often stated to be a rugged method requiring little sample preparation, the method is also sensitive and capable of detecting subtle changes, such as solid form transformations.^26,35^ Having the appropriate substance in its relevant solid form as reference or library spectra is important for the identification of an “unknown” sample. Because of this, the confidence of identification would be improved with an understanding of what solid forms can be expected in the precipitate. If a hydrate form does exist, formation of a hydrate can be expected in the precipitate from an aqueous solution. If that is the case, then a more robust identification would be based on a reference library containing Raman spectra of all possible solid forms. The structure of ceftriaxoneNa has been described;^36^ however, the calcium salt is not in the Cambridge Structural Database (CSD), but crystals have been identified from cases of kidney stones potentially also containing phosphate.^37,38^ Since ceftriaxoneCa was not commercially available, material for the reference spectra of ceftriaxoneCa was obtained by precipitating it from solutions of ceftriaxoneNa with calcium chloride dihydrate; however, this provides a reference of lower quality than desired.

**Figure 1:**
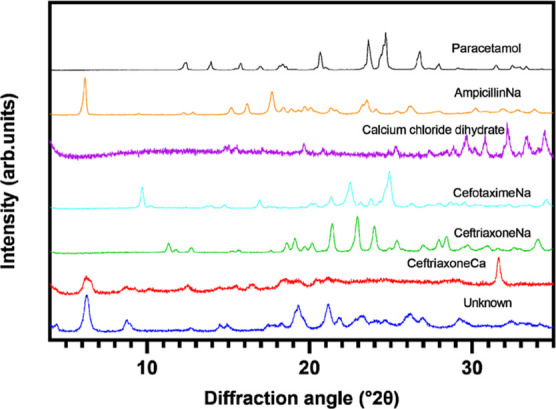
X-ray diffractogram of powder raw materials and the “unknown” precipitate from the combined drug mixture (metoclopramide and paracetamol were obtained as solutions and are not available).

The diffraction patterns from the XRPD analysis revealed that all drug products including the crystalline calcium chloride dihydrate as well as the “unknown” precipitated sample were at least partly crystalline (Figure 1). The XRPD pattern of the “unknown” sample has broad peaks and a higher baseline (halo), indicating that this sample contains smaller crystallite size and amorphous matter. The identification of solid form of the “unknown” sample is difficult based on the XRPD analysis because of the poor crystallinity of the sample. The crystalline or partly crystalline nature of the samples was confirmed in polarized light microscopy. Scanning electron microscopy (SEM) images of ceftriaxoneCa and the “unknown” precipitate are provided in the Supporting Information (Figure S1).

Thermograms from thermogravimetric analysis (TGA) showed that calcium chloride dihydrate lost 2–3% of the mass up to 100 °C and further heating resulted in a loss of 25% of the mass up to 165 °C, corresponding to a loss of 2 moles of water (Supporting Information Figure S2). The first weight loss is related to loss of free water, whereas the water of the dihydrate crystals is more tightly bound to the molecular structure and only leaves the crystal lattice at higher temperatures. The thermograms of the ceftriaxone salts and the “unknown” sample are more challenging to interpret as the samples were not carefully dried before analysis. The continuous weight loss that can be observed in all the three samples might also be influenced by samples being partially amorphous as indicated by the XRPD analysis and therefore renders a more hygroscopic behavior. The mass loss up to around 100 °C represents drying of the samples. The sodium salt of ceftriaxone has been described in the literature as a hemiheptahydrate,^36,39^ which could explain the weight loss in the range from 100 to 150 °C.

### 3.3 Identification of Precipitation in the Six-Component Mixture by Raman Spectroscopy

**Figure 2:**
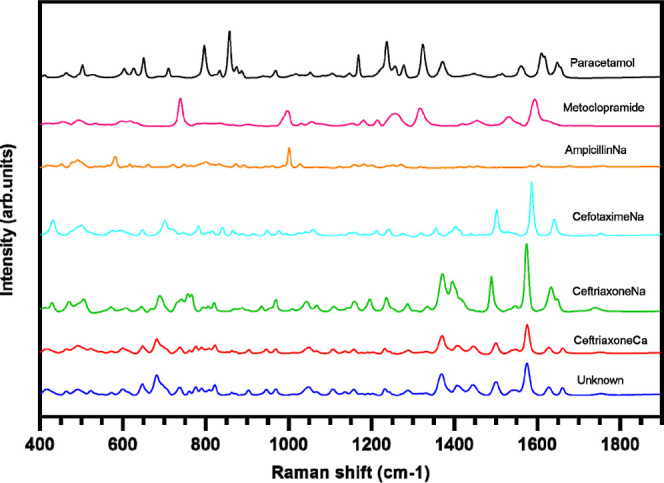
Raman spectra of the powder raw material of all drugs, ceftriaxone calcium, and the “unknown” precipitated from the combined drug mixture.

In Figure 2, the Raman spectra of all raw materials, ceftriaxoneCa, and the “unknown” are presented.

The Pearson correlation coefficients of the average “unknown” Raman spectrum against the spectra of the raw materials were calculated as the proportion of explained variance, *r*^2^, and is shown as a percentage. This was done in order to provide a rapid tool to identify the likely origin of the “unknown” precipitate at point of care. For ceftriaxoneCa, ceftriaxoneNa, and cefotaximeNa, the proportion of explained variance was 97, 48, and 8%, respectively (Figure 2). As expected, the correlation of the Raman spectra of the “unknown” samples with ceftriaxoneCa was high, while the correlation with ceftriaxoneNa and cefotaximeNa was significantly smaller. The proportions of explained variance for ampicillinNa, paracetamol, and metoclopramide were 2, 1, and 0%, respectively (Figure 2). This shows with high significance that the “unknown” sample was ceftriaxoneCa. In addition, if a reference spectrum of ceftriaxoneCa had not been available, from the correlation of the “unknown” to ceftriaxoneNa (48%), it could have been possible to predict that ceftriaxone was part of the precipitate, perhaps as a different salt or solid form. This could assist decision making at point of care in a potential emergency situation and help rule out which drug or drugs in a multi-drug co-administration had caused the precipitation. The precipitated drug could be eliminated from the multi-drug infusion and be administered in another i.v. catheter line.

The Pearson correlation coefficient does not require accurate baseline adjustment of the spectra and it is not sensitive to the variation in the relative intensity of the obtained spectra. It offers a fast way to provide a single number showing a comparison between a high number of spectra. In a setting where multiple drugs are present, which can precipitate into different salts, it could be used as a rapid tool to recognize the active substance related to the formation of the precipitate, and provided that the reference spectrum is available even identifies the specific salt that is formed under the clinical conditions.

**Figure 3:**
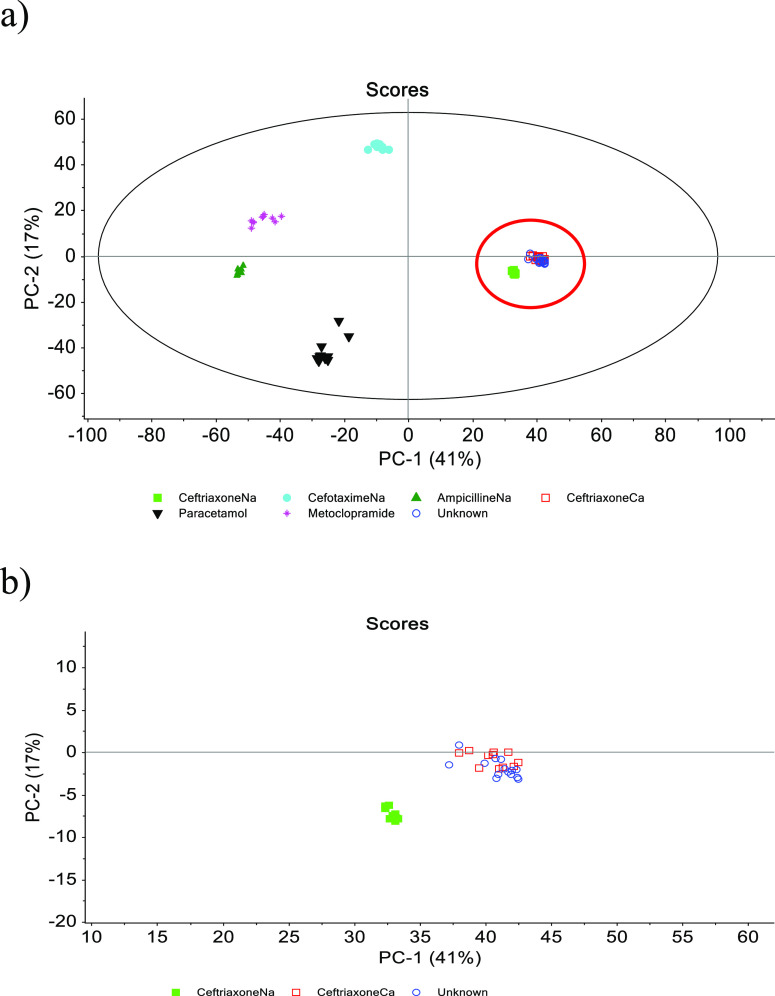
PCA of the Raman spectra (400–1900 cm^–1^) of all solid form drugs and the “unknown” precipitate from the multi-drug mixture. (a) Score plot. The red circle identifies the magnified area in (b) and (b) magnified area of the score plot separating ceftriaxoneNa, ceftriaxoneCa, and “unknown” sample.

Another way to obtaining an objective measure of similarity between Raman spectra would be to use multi-variate analysis. PCA is a recognized tool to identify correlations and grouping of similar samples in a large data matrix.^40^ PCA was performed on all the acquired Raman spectra of the range 400–1900 cm^–1^ for the “unknown” sample and each of the individual drug products (Figure 3). The first two PCs explained 58% of the variance in the data, and for these PCs, the score plot showed a close clustering of the “unknown” sample and ceftriaxoneCa and ceftriaxoneNa (red circle in Figure 3a). The difference between ceftriaxoneCa, ceftriaxoneNa, and the “unknown” samples as compared to the rest of the samples was mainly explained by the first principal component (PC1). The three were inversely related or very dissimilar to metoclopramide and ampicillinNa on PC1. The variation in the Raman spectral data in the direction of the second principal component (PC2) was not related to the “unknown” sample. PC2 mainly explained the variation between paracetamol and cefotaximeNa, as compared to the rest of the samples. Zooming in on the three samples in the red circle (Figure 3b), it is apparent that ceftriaxoneNa separated from the two other species with a slightly lower location in the PC1, PC2 space. The spectra of the “unknown” sample are similar to those of ceftriaxoneCa, and it can be noted that ceftriaxoneNa, the parent compound, is closer related to “unknown” and ceftriaxoneCa than any of the other drugs.

**Figure 4:**
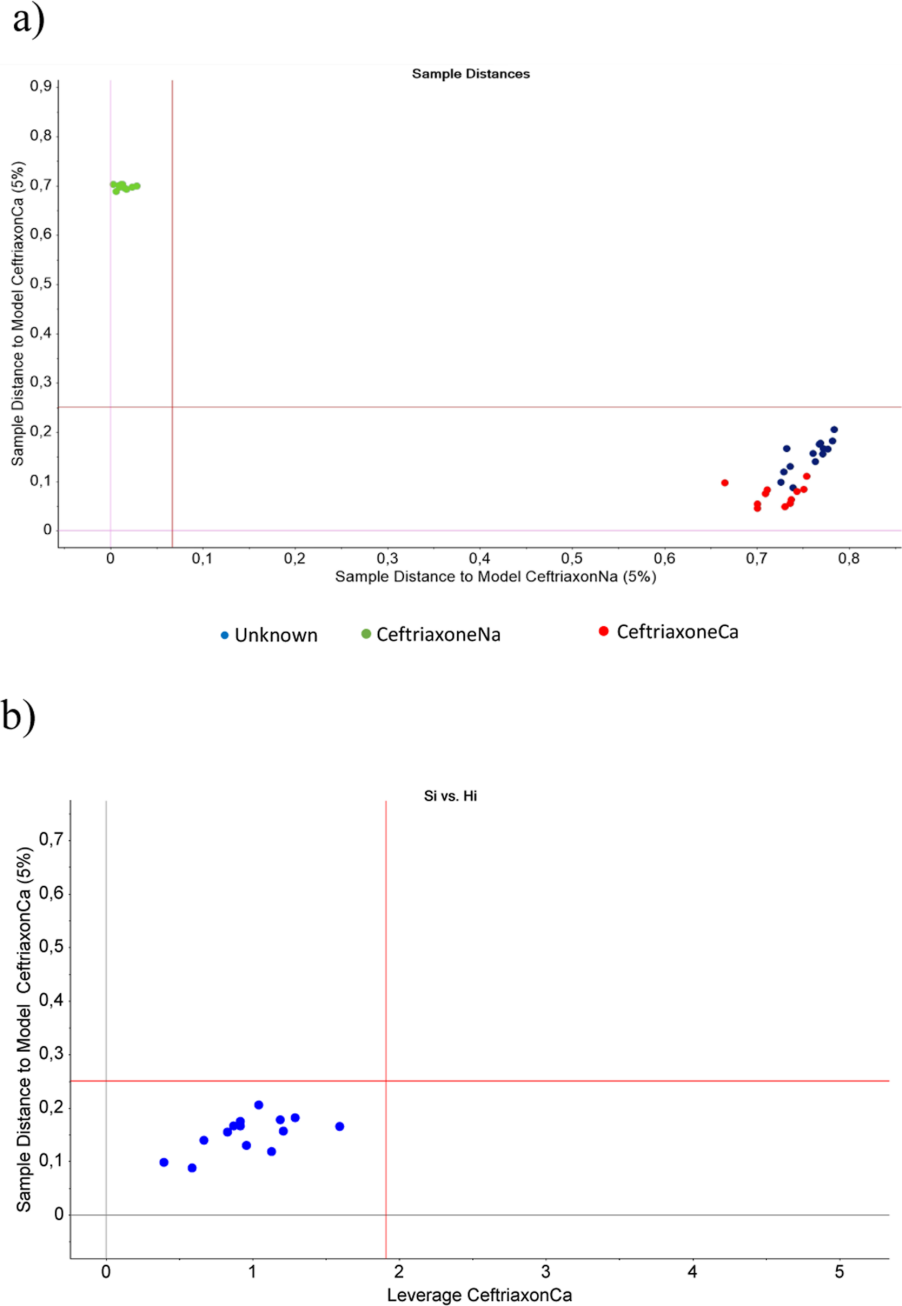
SIMCA for the classification of “unknown” sample: (a) Coomans’ plot showing “unknown” sample distance (blue) to the model of ceftriaxoneNa (green) and ceftriaxoneCa (red) and (b) membership plot showing classification of “unknown” (blue) with low leverage and low sample distance to the ceftriaxoneCa model. “Unknown” = classification samples. Red lines correspond to the 95% significance level.

Furthermore, a SIMCA classification based on wavenumbers from 400 to 1900 cm^–1^ was performed. All spectra captured of the “unknown” sample were classified as ceftriaxoneCa with a significance level of 95%. Both the Coomans’ plot (sample distances) in Figure 4a and the membership plot (*S*_i_ vs *H*_i_) in Figure 4b show that the “unknown” (classification samples) falls inside the limits of the ceftriaxoneCa model (significance level of 95%; red lines in Figure 4), indicating that the “unknown” precipitated sample is classified as ceftriaxoneCa and not ceftriaxoneNa. The main discriminators, which allowed the classification, were, as a function of their discrimination power (DP), the signals from 680, 1053, 1396, 1446, and 1489 cm^–1^ (Supporting Information, Figure S3). Comparing these wavenumbers to the spectra of ceftriaxoneNa on the one hand, and “unknown” sample and ceftriaxoneCa on the other (Figure 2), differences can be observed in the spectra in these wavenumber regions.

### 3.4 Identification of Sub-visible Single Particles by Raman Microscopy

For utilization of Raman spectroscopy in a clinical setting, the detection and identification of single sub-visual particles would be a great asset.^41^ To further explore such a possibility, the second Raman instrument, a HORIBA Jobin-Yvon, including a confocal microscope was used. A more simpler mix of drugs containing ceftriaxoneNa, ceftriaxoneCa, and calcium chloride dihydrate was investigated. All drugs were reconstituted in isotonic glucose (50 mg/mL) instead of water to adapt to a more clinically relevant situation, and the concentrations were optimized so that precipitation was scarce and slow. The drug mixture was filtered using a glass fiber filter to capture particles. The filtrates were dried prior to examination with the Raman microscope to search for particles that would be large enough to block capillaries and would pose a safety risk if infused into the body. The European Pharmacopoeia (2.9.19) consider particulate contamination of particles larger than 10 μm and 25 μm as undesired in marketed injectable drugs, and there are specified limits of numbers of particles per mL of these sizes.^42^

The smallest capillaries even have a diameter in the range of 5–8 μm,^43^ meaning that particles above this diameter may pose a patient risk.

**Figure 5:**
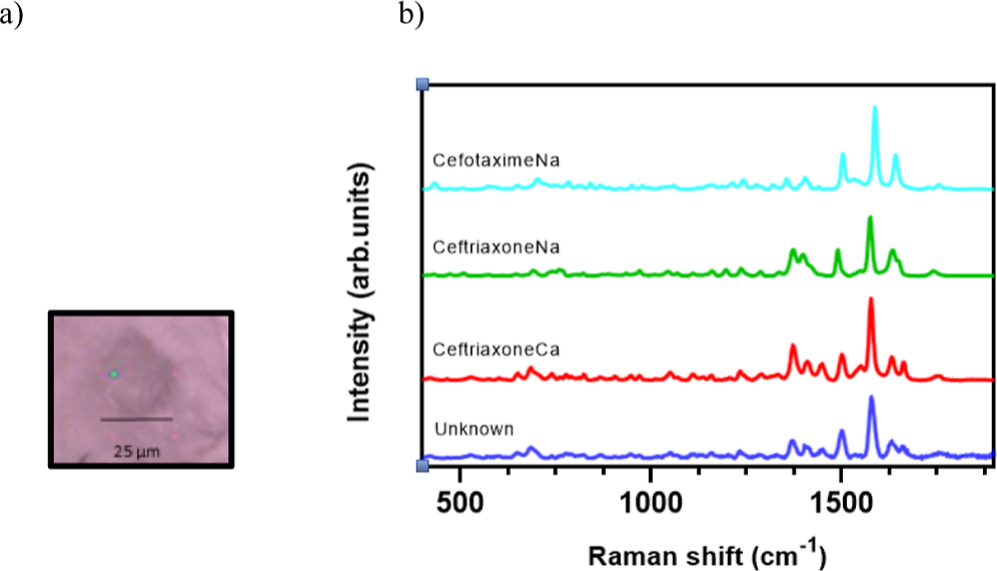
(a) Raman microscopy image of the “unknown” particle [blue color in (b)]. The green spot, which can be seen on the particle, is the focus point of the laser beam. (b). Raman spectra of the “unknown” precipitate from the mixture of ceftriaxoneNa, cefotaximeNa, and calcium chloride dihydrate together with the reference spectra of ceftriaxoneCa, ceftriaxoneNa, and cefotaximeNa.

As an example, a sub-visible particle (25 × 25 μm) was detected (Figure 5a) from the filtrate of the combined solution containing ceftriaxoneNa, cefotaximeNa, calcium chloride dihydrate, and glucose. The Raman spectrum of the particle was recorded and compared to the reference spectra of ceftriaxoneNa, ceftriaxoneCa, and cefotaximeNa, respectively (Figure 5b). The “unknown” particle spectrum was similar to that of ceftriaxoneCa, by visual comparison of the spectra. As in the spectra of the six-component multi-drug mixture (Figure 2), double peaks for the “unknown”, ceftriaxoneCa, and ceftriaxoneNa (merged) at 1600–1675 cm^–1^ were seen. The “unknown” is clearly not cefotaximeNa, which has a single peak at this wavenumber. The proportion of the explained variance between the spectrum of the sub-visible particle to the library spectra were 86, 52, and 33% for ceftriaxoneCa, ceftriaxoneNa, and cefotaximeNa, respectively. Again, using Pearson correlation calculations, it was possible to identify the most likely origin of precipitation as ceftriaxone, with ceftriaxoneCa being the most likely salt formed. The possibility to distinguish the identity of the “unknown” particle from other structurally similar antibiotics is very relevant in a clinical setting. It dictates which i.v. drugs are safe to co-administer in the same i.v. catheter line. As discussed above, sub-visible particles with sizes larger than the diameter of the smallest capillaries (>5 μm) could be trapped in the capillaries and pose a risk to the patient. It is therefore of relevance to be able to detect and identify sub-visual particles in a multi-drug mixture so the drug(s) that causes the problem and to advise not to co-administer these drugs/excipients together.

### 3.5 Raman Spectroscopy for Improved Compatibility Studies

With this study, we illustrate a potential application in identifying the molecular species involved in particle formation and precipitation from a multi-drug mixture. To pave the way for future in line detection of in situ precipitation caused by incompatibilities using Raman spectroscopy, we started simply but still retaining the complexity of compatibility testing. A model system of six drugs containing known incompatible substances was chosen to explore whether it would be possible to (1) determine the exact identity of the precipitate, (2) determine the origin of the compounds that formed the precipitating complex, and (3) whether it would be possible to do the identification based on a single particle. Another simplification was to study the precipitate in a dried state. Future studies should explore the use of Raman spectroscopy in a dynamic aqueous system that translates to the i.v. administration in patients. It is well known that the Raman spectrum of water is very weak and would normally not interfere with the spectra of the materials in solution or suspension. Raman spectroscopy has been reported for rapid qualitative and quantitative assessment of drugs in an aqueous solution^44^ and for in situ quality control of the solution of drugs in infusion bags.^45^ Furthermore, the use of Raman spectroscopy in process analytical technology emphasizes the feasibility of analyzing drugs in an aqueous environment.^23,46^ Therefore, implementing similar analyses in an aqueous environment in a clinical setting may not represent major scientific challenge. In this section, we attempt to look into the future and suggest potential application setups for using Raman spectroscopy for improved compatibility testing.

In this study, our model system was based on the case of lethal ceftriaxone calcium precipitation that lead to a warning issued by the US Food and Drug Administration (FDA) against the concurrent administration of i.v. ceftriaxone and calcium.^29^ Calcium precipitates have been found trapped in capillaries, which has caused embolism, resulting in infarction and vascular spasm and finally had led to several deaths. Nakai et al. investigated the precipitation of cefotaxime calcium and found that due to the formation of particles in the lower micrometer size region (when no precipitation could be visually observed), ceftriaxone should not be co-administrated together with calcium-containing products.^47,48^ On the other hand, parenteral lipid emulsions and lipid-containing parenteral nutrition (PN) are today formulated with an organic calcium salt (calcium gluconate), which makes the cation less active as compared to the inorganic calcium salts.^49,50^ Robinson and Sawyer summarized the compatibility of cephalosporins with lipid PN and found that ceftriaxone was compatible with those containing organic calcium.^51^ Using complexed calcium in the form of glucoronate reduces the free calcium ions available to react with ceftriaxone, which explains the finding that ceftriaxone was compatible. All clinicians today are well aware of not combining ceftriaxone with calcium. Nevertheless, our experience is that clinicians observe other precipitates in the infusion line, or there are syringe pumps triggering an alarm due to increased pressure. Critically ill patients do have the need for administration of numerous drugs and often have fewer venous access ports than needed drugs.

**Figure 6:**
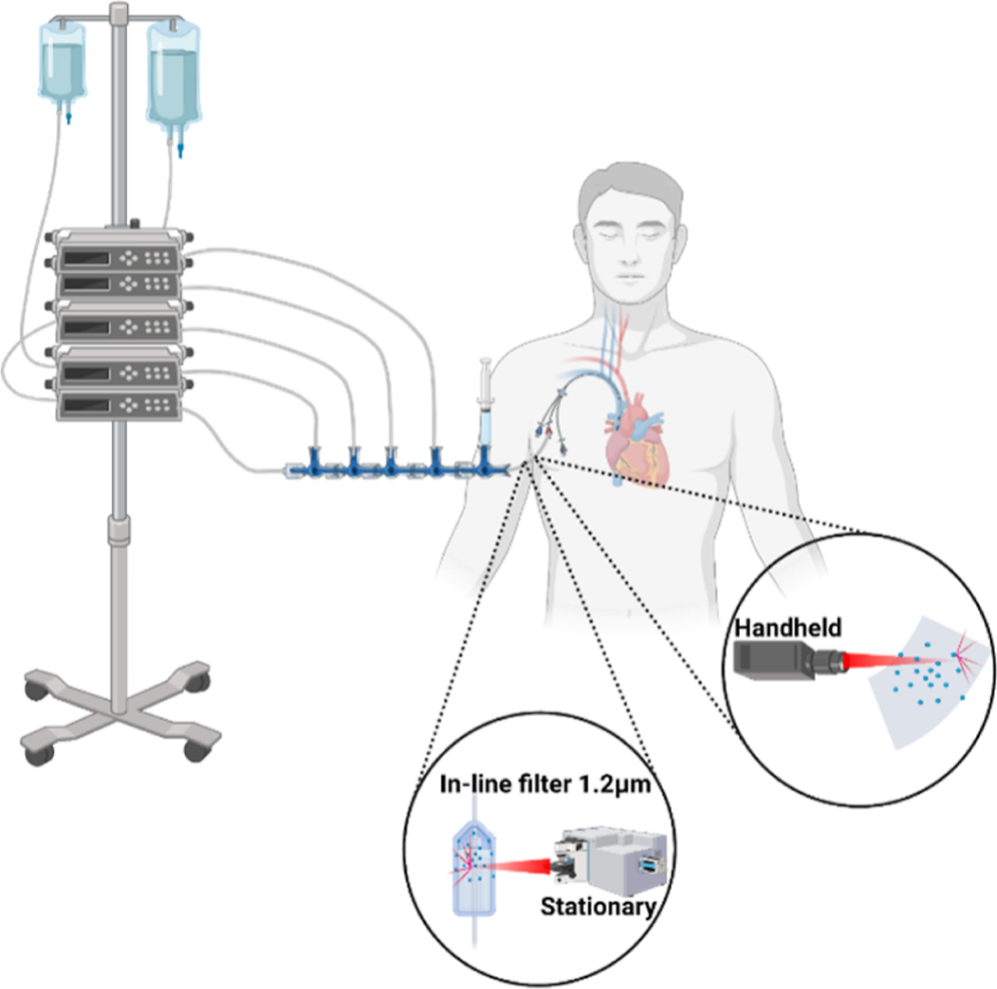
Potential future applications of Raman spectroscopy in the clinical setting analyzing clogged in-line filters with precipitated particles (left) or even in-line detection and identity proving of particles with a handheld instrument (right). Created with Biorender.com.

Based on these studies, various scenarios for the use of rapid Raman spectroscopy to solve clinically relevant compatibility issues can be envisioned. As mentioned above, non-destructive analyses of drug solutions inside infusion bags have been conducted using Raman spectroscopy, without perforation of the bag.^44,45^ This indicates that Raman detection of precipitation in infusion tubing is feasible without the need for perforation. The easiest approach is to implement a test laboratory or a facility in the hospital or in a hospital pharmacy that runs tests on drugs that are about to be co-administered in order to declare safe combinations by eliminating the compound(s) that can potentially precipitate. The library of Raman spectra from samples prepared according to an experimental plan can be built up from the precipitated samples for identification of the future cases. For new drugs, new combinations, or any other reason when a new combination therapy is desired, the relevant combinations can be quickly checked in the test laboratory and compared with the spectral library. The point-of-care testing would be a patient centric scenario where a handheld Raman probe is implemented for studying potential in-line particle formation (Figure 6). Handheld Raman instruments would be an attractive alternative for the point-of-care testing, but for this scenario to take place, ruggedness with regard to the characteristics of the sample to be identified by Raman is crucial. The use of filters that are often attached at the end of the infusion line when the compatibility of i.v. drugs is not known could also be explored (Figure 6). Particles on such filters are expected to be suitable for point-of-care analysis with a Raman instrument suitable for single-particle analyses. Either way, providing a rapid identity of this particulate material would be a valuable tool for subsequent safe drug administration and treatment consideration. It should be noted that some precipitates can be amorphous with a low-intensity Raman signal, or the compound of interest is simply a poor Raman scattering structure. This is underpinning the importance of development of robust Raman instruments with a possibility for using different laser sources with a different laser wavelength.

## 4 Conclusions

The precipitates observed in the hospital ward when incompatible drugs are combined are by the very nature of the process produced in an uncontrolled manner. Accurate and effective identification is of paramount importance when issues with precipitation are observed. In our study, we have shown that Raman spectroscopy is suitable for identifying the precipitated particulate species in a mixture of several drugs, both structurally similar and drugs that are therapeutically relevant for co-infusion. Using a single-particle Raman microscopic mapping of the filtered suspension with precipitate, even sub-visible precipitated particles with size around 25 μm could be detected and identified. Particles in this size range are a safety risk. This study demonstrates the potential of Raman spectroscopy in compatibility studies performed in a hospital setting.

## Supporting Information Available

SEM pictures of dried ceftriaxoneCa precipitated from ceftriaxoneNa and calcium chloride dihydrate; thermogram of powder raw materials of ceftriaxone sodium and calcium, calcium chloride dihydrate, and the “unknown” precipitate from the combined drug mixture; and SIMCA DP plot of the “unknown” sample against ceftriaxoneNa versus ceftriaxoneCa (PDF)
