# Supplementary material for: Co-administration of Intravenous Drugs: Rapidly Troubleshooting the Solid Form Composition of a Precipitate in a Multi-drug Mixture Using On-Site Raman Spectroscopy
Source: Mol Pharm. 2023 May 11;20(6):2853–63. doi: 10.1021/acs.molpharmaceut.2c00983 (PMC10245374; doi:10.1021/acs.molpharmaceut.2c00983)
Supplement: Supplementary file 1 — mp2c00983_si_001.pdf [file mp2c00983_si_001.pdf]

Supporting Information for Publication

Co-administration of intravenous drugs: Rapidly troubleshooting solid form composition of precipitate in a multi-drug mixture using on site Raman spectroscopy

*Niklas Nilsson[1, 2, *], Katerina Nezvalova-Henriksen[1, 2], Johan P. Bøtker[3], Niels Højmark Andersen[4], Bjarke Strøm Larsen[1], Jukka Rantanen[3], Ingunn Tho[1, *], Jørgen Brustugun[2, †]*

[1] Dept. of Pharmacy, University of Oslo, Oslo, Norway

[2] Oslo University Hospital and Oslo Hospital Pharmacy, Hospital Pharmacies Enterprise, South-Eastern Norway, Oslo, Norway

[3] Dept. of Pharmacy, University of Copenhagen, Copenhagen, Denmark

[4] Dept. of Chemistry, University of Oslo, Oslo, Norway

† deceased

* Corresponding authors:

Niklas Nilsson (niklas.nilsson@sykehusapotekene.no), Phone: +47 23073444

Ingunn Tho (ingunn.tho@farmasi.uio.no), Phone: +47 22844455

Department of Pharmacy, P.O. Box 1068, Blindern, 0316 Oslo, Norway

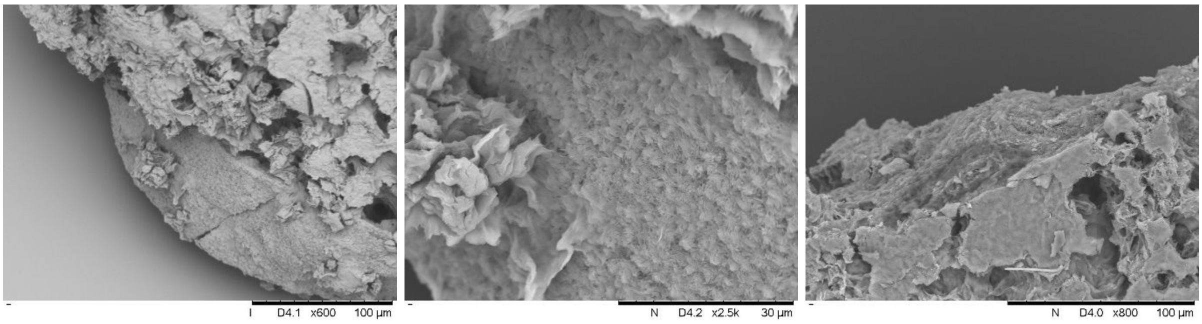

Figure S1: *SEM pictures of dried CeftriaxoneCa precipitated from ceftriaxoneNa and calcium chloride dihydrate. Sample has been washed and centrifugation in Eppendorf (all three pictures)*

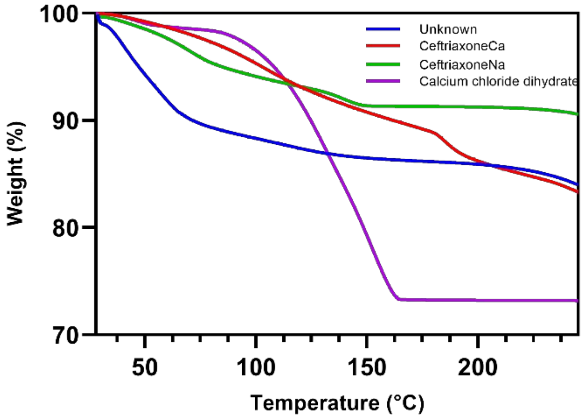

Figure S2. Thermogram of powder raw materials of ceftriaxone sodium and calcium, calcium chloride dihydrate and the "unknown" precipitate from the combined drug mixture.

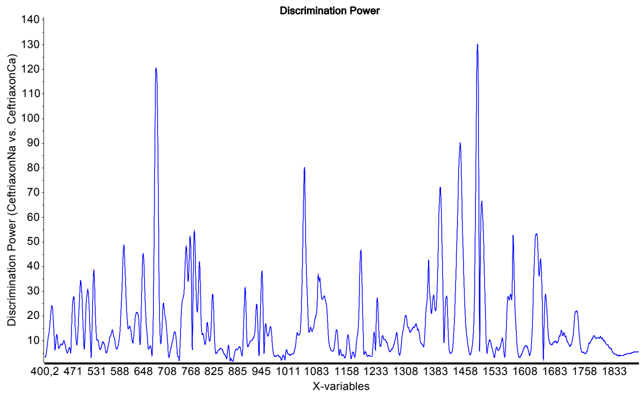

Figure S3: SIMCA discrimination power plot of "unknown" sample against ceftriaxoneNa versus ceftriaxoneCa.
